# MaxAlign: maximizing usable data in an alignment

**DOI:** 10.1186/1471-2105-8-312

**Published:** 2007-08-28

**Authors:** Rodrigo Gouveia-Oliveira, Peter W Sackett, Anders G Pedersen

**Affiliations:** 1 Center for Biological Sequence Analysis, Technical University of Denmark, Building 208, DK-2800 Lyngby, Denmark

## Abstract

**Background:**

The presence of gaps in an alignment of nucleotide or protein sequences is often an inconvenience for bioinformatical studies. In phylogenetic and other analyses, for instance, gapped columns are often discarded entirely from the alignment.

**Results:**

MaxAlign is a program that optimizes the alignment prior to such analyses. Specifically, it maximizes the number of nucleotide (or amino acid) symbols that are present in gap-free columns – the alignment area – by selecting the optimal subset of sequences to exclude from the alignment.

MaxAlign can be used prior to phylogenetic and bioinformatical analyses as well as in other situations where this form of alignment improvement is useful. In this work we test MaxAlign's performance in these tasks and compare the accuracy of phylogenetic estimates including and excluding gapped columns from the analysis, with and without processing with MaxAlign. In this paper we also introduce a new simple measure of tree similarity, Normalized Symmetric Similarity (NSS) that we consider useful for comparing tree topologies.

**Conclusion:**

We demonstrate how MaxAlign is helpful in detecting misaligned or defective sequences without requiring manual inspection. We also show that it is not advisable to exclude gapped columns from phylogenetic analyses unless MaxAlign is used first. Finally, we find that the sequences removed by MaxAlign from an alignment tend to be those that would otherwise be associated with low phylogenetic accuracy, and that the presence of gaps in any given sequence does not seem to disturb the phylogenetic estimates of *other *sequences.

The MaxAlign web-server is freely available online at http://www.cbs.dtu.dk/services/MaxAlign where supplementary information can also be found. The program is also freely available as a Perl stand-alone package.

## Background

A multiple alignment of nucleotide or protein sequences often forms the basis for phylogenetic analysis. In a perfect alignment, gaps correspond to insertion or deletion events, and as such should contain phylogenetic information on a par with substitutions. While some work has been done to make use of this type of data [1-3] there are still many unsolved issues. Additionally, gaps can also stem from misalignment, as well as from sequencing or data-management problems, in which case they obviously provide no useful information. Consequently, several bioinformatical and phylogenetic analyses are often based on alignments where gapped columns (i.e., columns containing at least one gap) have been discarded. For instance, removal of gapped columns is an option in the frequently used programs Paup [4], Paml [5] and Crann [6]. However, as the number of sequences in an alignment grows, the probability of having a gap in any given site also grows, and with it the risk of removing that site from the analysis. An alternative approach, that is sometimes used when applying maximum likelihood and other model-based methods, is to treat the gaps as unknown nucleotides (or amino acids) and sum over all the possible combinations, but this is not consensual and can become prohibitively costly for larger data sets. For some bioinformatical analyses, moreover, this alternative is not possible.

One way around this problem is to remove particularly gap-rich sequences, thereby ending up with a dataset containing more ungapped columns. This solution is of course not meaningful if the main goal of the analysis is to infer the topology of the phylogenetic tree connecting *all *the included taxa and one has a sufficiently long sequence. However, there are many other scenarios where the approach can be useful. For instance, it is often the case in molecular evolutionary analysis today that the focus is not on the phylogeny but on the analysis of the sequences themselves, and on properties of each position, such as the rate of evolution or the action of natural selection. In such cases keeping the sites in the analysis becomes important. The use of an automated, rapid alignment clean-up method is also clearly relevant in the case of large-scale or batch-type analyses, where phylogenies are produced from many potentially large data sets, or in the case of bioinformatical analyses not tolerant to the presence of gaps.

## Results and discussion

### Overview

The goal of this tool is to maximize the alignment area, defined as the number of characters that are present in gap free columns. Alignment area is thus equal to the number of sequences included in the alignment times the number of columns that have no gaps. This maximization of the alignment area is done by selectively removing sequences from the alignment. Finding the right sequences to remove, however, is not a straightforward problem (see Methods for details on the MaxAlign algorithm).

To scrutinize the performance of MaxAlign, we have analyzed very large sets of protein alignments from two different sources: (1) alignments extracted from the Pfam database[7], and (2) synthetic alignments created by simulating evolution on a set of three different trees using the program simprot [8]. The Pfam data were chosen to be representative of typical, fairly diverged biological alignments, and consisted of all the 5242 alignments from Pfam-A, release 21.0, that had between 30 and 500 sequences. The simulated data were constructed so it resembled the Pfam-data in terms of sequence divergence, and in the number and length of gaps (see below). Specifically, we analyzed the following aspects of MaxAlign behaviour: (1) performance of the heuristic version of the MaxAlign algorithm (optimality and computation time), (2) Improvement of alignment area by MaxAlign, (3) Ability of MaxAlign to remove "contaminating" (non-homologous) sequences from an alignment, (4) Impact of MaxAlign on phylogenetic accuracy (meaning how closely the reconstructed tree resembles the true tree), and (5) Impact of MaxAlign on the computation time of subsequent maximum likelihood phylogenetic analysis.

### Performance of MaxAlign heuristic algorithm

MaxAlign can use one of two different algorithms: a heuristic algorithm (which is faster, but which is not guaranteed to find the best solution), and an optimal algorithm (which is an adaptation of branch-and-bound; see Methods). We tested both algorithms on the full Pfam dataset. The average runtime for the heuristic was 0.6 seconds per alignment (user time) demonstrating that use of MaxAlign is not a bottleneck for data analysis. Among the 5242 alignments, the heuristic algorithm found the optimal solution in 78% of the alignments. In only 4% of the alignments, the heuristic algorithm did not find the optimal solution. The remaining alignments either could not be improved from the start (4%), or the branch and bound algorithm could not reach a solution in 2 hours (15% of the alignments). Thus, the heuristic algorithm found the best solution in 95% of the cases in which a solution was found. Moreover, analysis of the solutions in the few cases where the heuristic was not optimal showed that the alignment area of the heuristic solution was, on average, 99.0% of the optimal solution (median: 99.6%). On the whole, we therefore recommend using the heuristic algorithm of MaxAlign.

### Improving the alignment area

The alignment area of the Pfam alignments was quite variable, with a majority (52.2%) having no gap free columns (and consequently an alignment area of 0). The average number of gap free columns in the unprocessed alignments was only 19.4, and the average alignment area 1494 (see table 1). The optimization of alignment area performed by MaxAlign resulted in a 10-fold increase to an average alignment area of 16000, corresponding to an 8-fold mean increase of 143.2 gap free columns per alignment, at the average cost of only 19% of the sequences in the alignment. In a small minority (4%) of the 5242 cases it was not possible to improve the alignment area by removing sequences, and MaxAlign therefore had no effect. Thus, the majority of Pfam alignments were unusable for analyses requiring the elimination of gapped columns and became usable datasets by processing with MaxAlign.

**Table 1:** Changes in alignment dimensions caused by MaxAlign

	**Original**	**MaxAlign**
Alignment area	1494	16000
Number of gap-free columns	19.4	162.6
Number of sequences	139.8	112.0

Average values for alignment dimensions before and after processing with MaxAlign. Estimated from 5242 Pfam alignments with between 30 and 500 sequences.

### Removing non-homologous sequences

It is not rare that data sets are contaminated with non-homologous sequences. These will typically result in the generation of many gaps when the sequences are aligned. To investigate how well MaxAlign performs in removal of such contaminating sequences we therefore constructed a set of noisy datasets: for each of the 5242 original Pfam alignments we added random, non-homologous sequences (taken from other Pfam data sets) such that the final content of noise was approximately 10%. These datasets were then re-aligned using the program mafft with default parameters[9]. We then ran MaxAlign (heuristic algorithm) on the resulting alignments and analyzed the performance in terms of removal of contaminating sequences. We found that, taken over all 5242 alignments, MaxAlign performed very well, removing on average 87% of the non-homologous sequences per alignment (median removal success: 96%). In 46% of all cases, MaxAlign was able to remove all non-homologous sequences from the alignment.

### Impact on phylogenetic analysis

#### Accuracy

**Figure 1:**
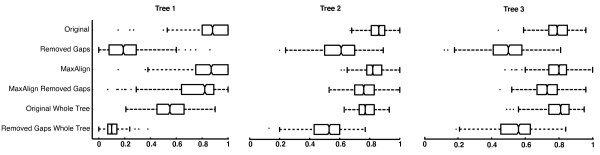
**Accuracy in phylogenetic inference**. Comparison of phylogenetic accuracy obtained with different data sets. Accuracy is measured as tree similarity between the true tree (used for simulating the data set) and the reconstructed tree. Each line shows the distribution of the accuracy results from 1000 different data sets, in the form of a box plot. The box has lines at the lower quartile, median and upper quartile. The whiskers extend from each quartile to the most extreme values within 1.5 times the interquartile range. Outliers falling outside this range are marked with dots. The datasets are in the same order (from top to bottom) as in table 2: The top two rows show the original dataset without and with removal of gapped columns, respectively. The third and fourth rows show the equivalent MaxAlign datasets. The trees in the top four rows are being evaluated on the subset of sequences shared by all data sets ("Subset"), while the lower two rows show the results for original datasets when evaluated on the full set of sequences ("All").

It is an unsolved issue in phylogenetic analysis how to best deal with gaps. We touched upon this topic by investigating how MaxAlign and removal of gapped columns interfere with phylogenetic tree inference. To do so, we first simulated 1000 alignments along each of 3 trees of different shapes. From each of these synthetic alignments, we then constructed three derived data sets by (a) removing gapped columns, (b) applying MaxAlign, and (c) applying MaxAlign and removing gapped columns. For each of the resulting 12,000 synthetic alignments (3,000 original plus 9,000 derived ones), we then performed maximum likelihood phylogenetic analysis using the program phyml. The accuracy of phylogenetic reconstruction was then analyzed by calculating the normalized symmetric tree similarity (see Methods) between the true tree (used to simulate the alignments) on one hand, and the four trees estimated by phylogenetic analysis on the other. These tree similarities were computed based on the subset of taxa shared by the original and maxaligned datasets. For the trees inferred from the original (simulated) alignment and from the original alignment excluding gaps, we also computed the similarity to the true tree based on the full set of taxa. The outcome of this comparison can be found both in figure 1 and table 2.

**Table 2:** Effects of MaxAlign and removal of gapped columns on phylogenetic accuracy

**MaxAlign**	**Removal of gaps**	**Set of taxa used for comparison**	**Tree 1**	**Tree 2**	**Tree 3**
-	-	Subset	85.9 (0.5)	85.4 (0.2)	78.7 (0.3)*
-	+	Subset	21.0 (0.5)	60.1 (0.4)	49.1 (0.4)
+	-	Subset	82.8 (0.6)	82.3 (0.2)	78.6 (0.3)*
+	+	Subset	74.6 (0.7)	75.9 (0.3)	72.4 (0.3)
-	-	All	56.1 (0.5)	78.4 (0.2)	79.7 (0.3)
-	+	All	10.3 (0.2)	52.7 (0.4)	53.2 (0.4)

Phylogenetic accuracy for datasets with/without removal of gapped columns, and processed/not processed by MaxAlign. Accuracy is measured as the average normalized symmetric tree similarity between the true tree (used for simulating data) and the individual inferred trees, with the standard error of the mean (in %) given in parenthesis. "Subset" refers to the set of taxa (sequences) common to the original and the MaxAligned data. "All" means all taxa in the original data set. Values marked with * are the only ones whose difference is not statistically significant.

When measuring phylogenetic accuracy on the subset of taxa that remain after MaxAlign processing, it can be seen that the highest phylogenetic accuracy was achieved by using all the sequences available without discarding gapped columns (figure 1, all rows but 2^nd ^and last). However, it can also be seen that applying MaxAlign decreases the accuracy only very slightly (figure 1, compare "Original" and "MaxAlign"). Moreover, if one decides to discard columns with gaps, then using MaxAlign is clearly the best option (figure 1, compare "Removed Gaps" with "MaxAlign Removed Gaps").

If the accuracy of trees (based on non-MaxAligned data) is measured on the *full *set of taxa, then a different trend is apparent: especially in trees 1 and 2, it can be seen that processing the alignment with MaxAlign increases the accuracy of the phylogeny (figure 1, accuracy on full set of taxa is shown in bottom two rows; compare "Original Whole Tree" to "MaxAlign"). The reason for this phenomenon is that MaxAlign predominantly removes sequences with many gaps. These will necessarily also be the ones having the fewest amino acids, and therefore the ones associated with the highest phylogenetic uncertainty. It is important to note that in phylogenetic analyses that measure support for each branch, such as Bayesian analysis or Bootstrap, the position of these taxa would show up with low support values. It should also be noted that the presence of gaps in some sequences does not seem to disturb phylogenetic inference on the remaining sequences (figure 1, compare "Original" to "Original Whole Tree"). It should be noted that we have not investigated the additional impact of alignment error on this, since we directly use the simulated (and therefore perfect) alignments themselves.

#### Computation time

One reason for discarding gapped columns is that summing over all possible missing values is a time consuming step in a maximum likelihood calculation. We have evaluated how much time is saved by using MaxAlign with and without discarding columns with gaps. The results are presented in table 3.

**Table 3:** Runtimes of the phylogenetic analysis

**MaxAlign**	**Removal of gaps**	**Mean**	**Median**	**Maximum**
-	-	17.7	8.4	408
+	-	8.1	5.5	78
+	+	4.8	3.7	50

Runtimes of the phylogenetic analysis. All times measured in minutes. Both application of MaxAlign and removal of gapped columns resulted in strongly decreased runtimes.

It can be seen that either using MaxAlign or removing columns with gaps nearly halves the computer time needed to find the phylogenetic tree. Removing columns with gaps does so by preventing the summation over unknown characters as well as reducing the number of sites to be included in the analysis. MaxAlign diminishes the number of summations required and also reduces the size of the dataset, by including fewer sequences.

The computer time required for these phylogenetic analyses was particularly short, as we used a very fast program (phyml), and the alignments contained few sequences. In situations using more time-consuming analyses and software, as well as larger alignments, these relative differences in computing time will have an increased impact.

## Conclusion

In the present work, we have shown how MaxAlign is helpful in "cleaning up" big alignments, and demonstrated how it may be used in connection with phylogenetic analysis. In the analysis presented above, we found that the use of MaxAlign more than halved the running time of the analysis even when gapped columns were not excluded. When gapped columns were discarded from the alignment, the output was of much higher quality if MaxAlign was first used to pre-process the data, as the number of columns included in the analysis increased immensely. We have also shown that in the conditions tested, the accuracy of the phylogenetic estimate of the tree topology increases if one includes the gapped columns. The sequences removed by MaxAlign were predominantly those associated with the highest degree of uncertainty regarding their placement in the final trees.

The use of MaxAlign prior to bioinformatical and phylogenetic analyses optimizes the number of nucleotides or amino acids present in ungapped alignment columns (the "alignment area"), thereby increasing the amount of useful data and/or the speed of the analysis. Moreover, MaxAlign is also very helpful in detecting misaligned or defective sequences without requiring manual inspection. Given its very short running times and ease of use, it can without difficulty be used as a step in both automated and manual phylogenetic and bioinformatical analyses.

## Methods

### MaxAlign

**Figure 2:**
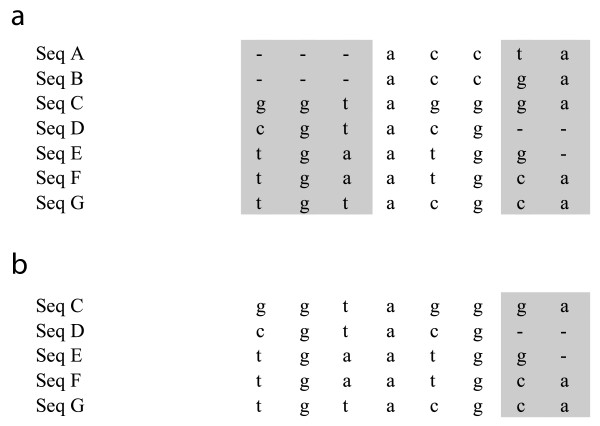
**Example of MaxAlign processing**. Example alignment, before (a) and after (b) MaxAlign. In the original unprocessed alignment (a), only the three middle columns would be included in a subsequent analysis (alignment area = 3 rows × 7 columns = 21). The first three columns have the same gap pattern. After MaxAlign processing (b) (resulting in removal of sequences A and B) only the last two columns would be excluded by having gaps (alignment area = 5 rows × 6 columns = 30).

MaxAlign maximizes the alignment area of an alignment by removing selected sequences. It has two algorithms to do so: one heuristic and one branch-and-bound. Both are based on the concept of *gap pattern*. In the example alignment shown in figure 2, one can see that the first three columns have the same gap pattern, namely the presence of gaps in the first two lines (corresponding to sequences A and B). All columns sharing that gap pattern would yield ungapped columns if sequences A and B were removed. For every gap pattern there is therefore a set of sequences requiring removal to produce ungapped columns. The heuristic algorithm works by first determining the sequence set for each gap pattern and then iteratively removing the set of sequences that most increases the alignment area per sequence removed, until it reaches the solution with the highest absolute alignment area (shown in figure 2b). The heuristic algorithm is always used in MaxAlign. The branch-and-bound algorithm, which is guaranteed to find the best possible solution at the expense of a longer running time, can optionally be used to check if the heuristic algorithm has found the optimal solution.

MaxAlign has several additional features. For example, it is possible to select a subset of sequences that should be kept in the final alignment, even if their removal would yield an improvement of the alignment area. This is done by simply adding a plus sign ("+") in front of the sequence name in the fasta file. In this way, the user can preserve key sequences in his/her analysis. Also, users interested in codon-based analyses can run MaxAlign on protein sequences and obtain their output alignments both in protein and nucleotide format (as long as they also provide the corresponding nucleotide sequences).

The server is freely accessible at [10] where a stand-alone Perl version can also be downloaded free of charge [see Additional file 1]. The input to MaxAlign is an alignment in fasta format (two in the case of codon-based analyses). The main output is an alignment (in fasta format) consisting of the set of sequences that maximizes the alignment area. The program also lists the sequences that were included and excluded from the original alignment, and furthermore provides a report on the resulting improvement.

### Improving Alignment area

To test the relevance of MaxAlign in real biological problems, the Pfam-A Release 21.0 of full alignments [7] was used. All the 5242 alignments comprising between 30 to 500 sequences were submitted to MaxAlign. The mean number of sequences in this set of alignments was 140 (median:90). The alignment area and number of ungapped columns was recorded, before and after MaxAlign. In this analysis, as in all of the following unless otherwise stated, the heuristic algorithm was used.

### Removal of non-homologous sequences

To test the ability of MaxAlign in removing non-homologous sequences from an alignment, we added noise sequences to each of the 5242 above-mentioned Pfam alignments in a ratio of 1:10. Thus, the alignments ended up containing between 33 to 550 sequences. These alignments were submitted to MaxAlign and the fraction of noise sequences removed was recorded. For each alignment, non-homologous sequences were chosen randomly from the rest of Pfam.

### Impact on phylogenetic analysis

#### Accuracy

To test the impact of MaxAlign in phylogenetic analyses, a set of benchmarking phylogenetic analyses was set up. To be certain of the true tree topology, we simulated alignments along known trees, using simprot [8]. To ensure these alignments were similar to biological alignments, we estimated several parameters from the Pfam alignments having between 30 and 100 sequences (a total of 2416). Specifically, we estimated the mean length of sequences (with and without gaps), the distributions of both length and number of the indels, and the gamma shape parameter on the distribution of evolutionary rates among sites (by performing a pre-phylogenetic analysis on each of these alignments). Parameters in simprot were then tuned in order to obtain similar distributions and statistics on the simulated alignments. Specifically simulations were performed using the following parameter settings: -r 180 -g 0.01 -b 4 -c 0.2 -t 2 -x 1.8. For each tree, 1000 alignments were simulated (data on the simulated alignments is shown in table 4).

**Table 4:** Data on the simulated datasets

	**Tree 1**	**Tree 2**	**Tree 3**	**Pfam**
Average sequence identity	19%	30%	42%	-
Alignment length	1080	629	597	404
Sequence length	173	177	169	171
Original number of sequences	32	33	46	-
Average number of sequences after MaxAlign	14.1	22.6	28.8	-
Average number of indels per sequence	66.6	54.3	48.5	32
Average length of indels	13.6	8.3	8.8	7

Description of the simulated alignments used for testing the accuracy of phylogenetic inference with MaxAlign and removal of gapped columns, as well as the Pfam estimates used to tune the simulation parameters.

**Figure 3:**
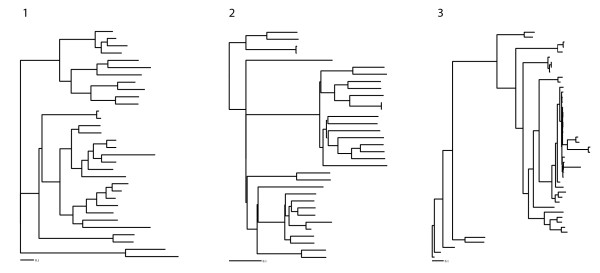
**Tree topologies used to simulate alignments**. The trees used to simulated the alignments. From 1 to 3: TF101002, TF101523 and TF105969.

The 3 trees chosen to generate the simulated alignments had very different kinds of topology, and were downloaded from the TreeFam database[11]. Tree 1 was TF101002, a 32-taxa tree of Cyclin A, with evolution occurring mostly near the tips; Tree 2 was TF101523, a 33-taxa tree of the Eukaryotic translation initiation factor 3, subunit 6 interacting protein, with evolution occurring throughout the tree; Tree 3 was TF105969, a 46-taxa tree of the tumour rejection antigen gp96, with evolution occurring mostly at the base of the tree (see figure 3).

Each simulated alignment gave rise to 4 derived datasets: the alignment after processing with MaxAlign, but keeping the gapped columns, the alignment after processing with MaxAlign and removal of gapped columns, the original alignment with the gapped columns removed and the original alignment without any processing.

These datasets were then used as the basis for reconstructing the tree topology. This was done by using phyml [12] with the WAG substitution model and having the evolutionary rate among sites modelled by a gamma distribution discretized into 4 categories, with the shape parameter being estimated from the data. The tree topologies found were then compared among the different datasets, using Normalized Symmetric Tree Similarity (see below). As MaxAlign removes sequences from the alignment, the resulting trees have a smaller set of leaves. We therefore compared the correct tree against the four tree topologies resulting from the phylogenetic analyses using only the subset of taxa that remained after MaxAlign processing. For the two trees based on data that had not been processed by MaxAlign, we furthermore compared to the true tree using the full set of taxa. Statistical significance of differences between similarities was assessed using a double-sided t-test at 5%.

#### Computation time

All Pfam alignments having between 30–100 sequences (a total of 2416) were included in this analysis, which consisted of measuring the average duration of phylogenetic analyses, in a way very similar to the one described above, apart from using real alignments instead of simulated alignments (same dataset generation, same phylogenetic analysis). We did not analyze computation time on the original datasets (those not processed by MaxAlign) with removal of gapped columns, as the majority of these alignments had zero columns available for the analysis.

#### Comparing the two MaxAlign algorithms

To compare the performance of the two algorithms available in MaxAlign, we applied them to the full set of 5242 Pfam alignments described above and evaluated the solutions in terms of time spent and changes in alignment area.

#### Normalized Symmetric Tree Similarity

The degree ofdissimilarity between two phylogenetic trees can be quantified using a number of different measures of tree distance. The most widely used measures are probably the symmetric distance[13], the quartets distance [14], and the branch score distance [15]. The two former measures focus on tree topology, while the latter also uses branch length information. Here, we propose a simple measure of (topological) tree similarity that is based on the symmetric distance of Robinson and Foulds, and that can be used to compare tree similarities measured on different sets of taxa.

Any branch in a phylogenetic tree can be thought of as defining a bipartition where the leaves are divided into those present on one side of the branch and those present on the other side of the branch. The symmetric distance of Robinson and Foulds is simply the number of bipartitions (branches) in tree 1 that are not present in tree 2 plus the number of bipartitions in tree 2 that are not present in tree 1. This is an intuitively understandable measure of treedissimilarity that is fairly rapid to compute.

We now propose a normalized version of this distance measure: simply divide the symmetric distance between two trees by the total number of bipartitions present in the two trees. (The total number of bipartitions in the two trees is used for normalization since it is the maximum possible symmetric distance). This "Normalized Symmetric Distance" is zero when trees are identical and 1.0 when they have absolutely no bipartitions in common. From the Normalized Symmetric Distance (NSD), the corresponding "Normalized Symmetric Similarity" (NSS) can be computed simply as NSS = 1.0 – NSD. This similarity measure is 1.0 for identical trees and zero for trees without any common bipartitions.

## Authors' contributions

RGO had the idea for the project, designed a first version of MaxAlign and wrote the paper. PWS designed the current algorithms and implemented all the final software. AGP wrote the paper, designed and implemented all tests and supervised the project. All authors read and approved the final manuscript.

## Supplementary Material

Additional file 1 MaxAlign perl script. The file contains the MaxAlign perl script that can be used at the command line. Click here for file
